# Supplementary material for: Altered tRNA processing is linked to a distinct and unusual La protein in Tetrahymena thermophila
Source: Nat Commun. 2022 Nov 28;13:7332. doi: 10.1038/s41467-022-34796-3 (PMC9705548; doi:10.1038/s41467-022-34796-3)
Supplement: Supplementary file 1 — Supplementary Information [file 41467_2022_34796_MOESM1_ESM.pdf]

Supplementary Information for "Altered tRNA processing is linked to a distinct and unusual La protein in *Tetrahymena thermophila*"

Kerkhofs, Kyra[1]; Garg, Jyoti[1]; Fafard-Couture, Étienne[2]; Abou Elela, Sherif[3]; Scott, Michelle S.[2]; Pearlman, Ronald E.[1]; Bayfield, Mark A.[1*]

[1] Department of Biology, Faculty of Science, York University, Toronto, Ontario M3J 1P3, Canada,

[2] Département de Biochimie et de Génomique Fonctionnelle, Faculté de Médecine et des Sciences de la Santé, Université de Sherbrooke, Sherbrooke, Québec J1E 4 K8, Canada,

[3] Département de Microbiologie et d'Infectiologie, Faculté de Médecine et des Sciences de la Santé, Université de Sherbrooke, Sherbrooke, Québec J1E 4 K8, Canada.

[*]Correspondence: bayfield@yorku.ca (M.A.B.)

Supplementary Figure S1. Multiple sequence alignments of La proteins from several eukaryotes demonstrate high conservation of the LaM and absence of the RRM1 in alveolates.

Supplementary Figure S2. Mlp1 demonstrates preferential binding of certain tRNA isotypes and unprocessed pre-tRNAs.

Supplementary Figure S3. TGIRT-sequencing replicates reproducibility determined by correlation plots.

Supplementary Figure S4. Mlp1 binding to typical La protein target RNAs.

Supplementary Figure S5. Mlp1 binding to uridylate RNA does not discriminate between the position of the uridylate.

Supplementary Figure S6. tRNA mediated suppression protein expression in *S. pombe*.

Supplementary Figure S7. Confirmation of the partial Mlp1 *T. thermophila* knockout strain.

Supplementary Figure S8. The effect of short 3'-trailer sequences on 5'-leader composition and Mlp1 binding affinity.

Supplementary Figure S9. Primary sequence alignments of the 3'-exonuclease Rex1p, 3'-endonuclease RNase Z / ELAC2 and LSm2-8 complex from different eukaryotic species.

Supplementary Table S1. Kd values from EMSAs determining binding of Mlp1 to different processed pre-tRNA intermediates and mature tRNA.

Supplementary Table S2. Summary of 3'-trailer length in different eukaryotic species calculated using different RNA polymerase III termination signals.

Supplementary Table S3. NCBI accession numbers of primary amino acid sequences used for conservation analysis.

S1

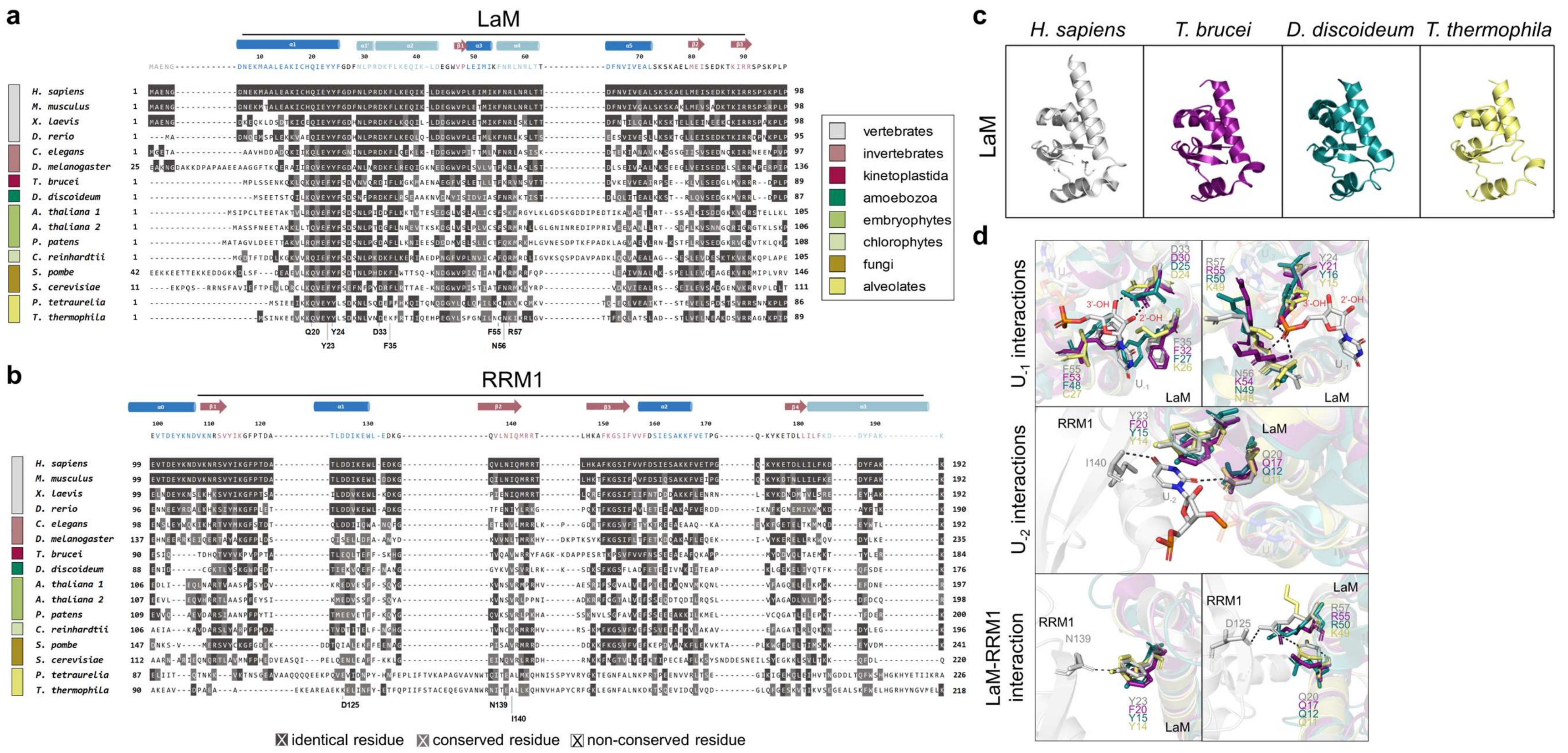

Figure legend on next page

Supplementary Figure S1. Multiple sequence alignments of La proteins from several eukaryotes demonstrate high conservation of the LaM and absence of the RRM1 in alveolates.

a,b Primary amino acid alignments of full RNA-binding domains La motif (LaM) (**a**) and RNA recognition motif-1 (RRM1) (**b**) from different eukaryotic lineages. A dark grey background indicates identical residues, light grey conserved residues and white indicates no conservation. Most uridylate binding residues are conserved in the LaM of alveolates, whereas residues in the RRM1 are more variable. Important uridylate binding residues are shown at the bottom. The secondary structure motif of the high-resolution hLa protein structure is shown at the top with β-sheets shown in red and α-helices in blue (dark blue: typical α-helices found in the winged-helix fold and classic RRM, light blue: inserted α-helices found in La proteins specifically) (PDB: 2VOD). **c** High-resolution structures of the LaM in different species: *H. sapiens* in white (PDB: 2VOD)[1], *T. brucei* in purple (PDB: 1S29)[2], *D. discoideum* in teal (PDB: 2M5W)[3] and the predicted tertiary structure *for T. thermophila* in yellow[4]. **d** Magnified views of La-U_{-1}, La-U_{-2} and LaM-RRM interactions. U_{-1} is the most 3'-terminal uridylate possessing 2'-OH and 3'-OH ends. Most uridylate binding residues located in the LaM are conserved in *T. thermophila,* except for F35 and F55 (human La numbering) which bind the most 3'-terminal U_{-1}. Carbon atoms within the RNA (U_{-1} and U_{-2}) from the *H. sapiens* La structure are colored in white with other atoms colored by type: oxygen in red; nitrogen in blue and phosphorous in orange. Hydrogen bonds are shown as black dashed lines.

S2

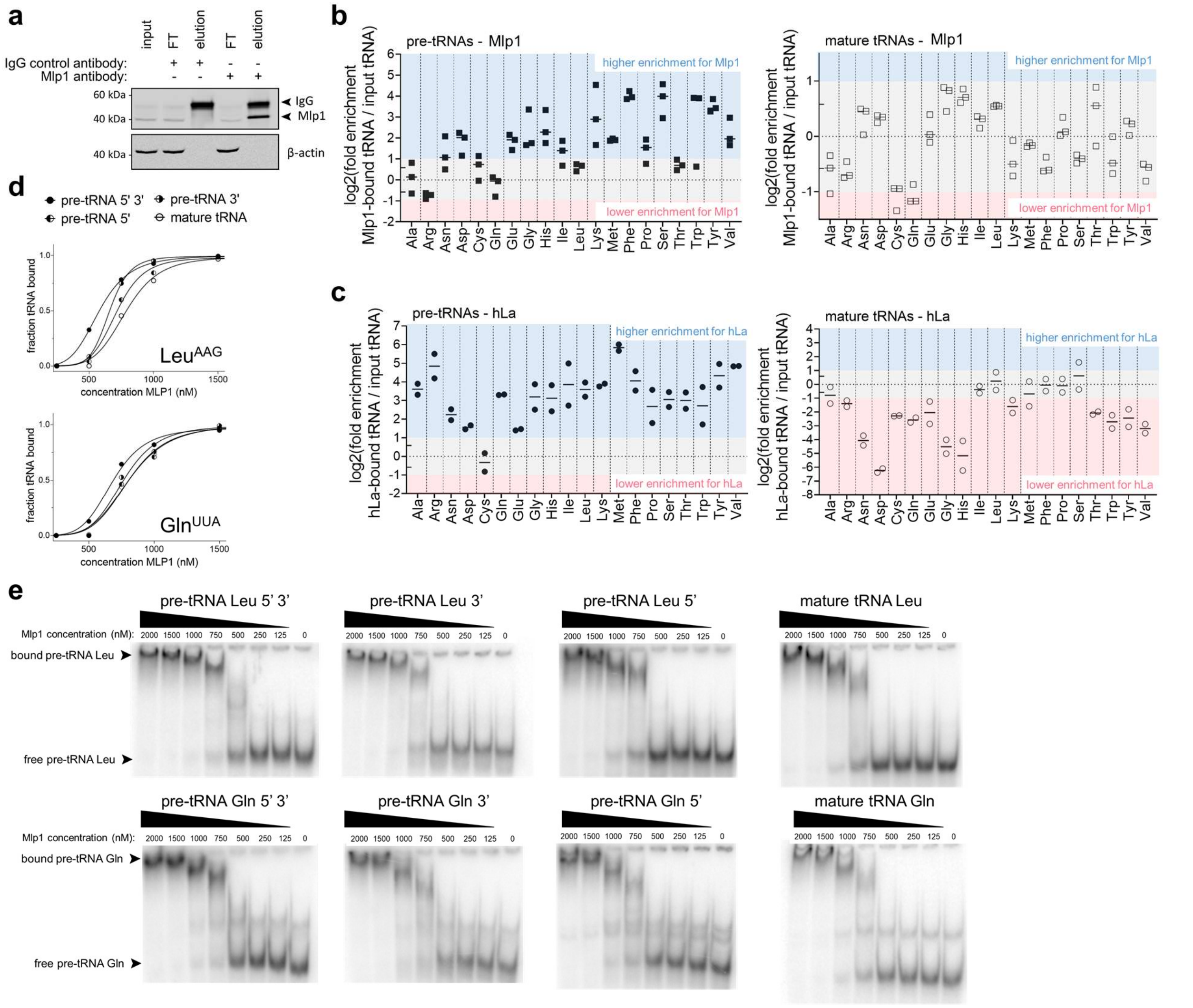

Supplementary Figure S2. Mlp1 demonstrates preferential binding of certain tRNA isotypes and unprocessed pre-tRNAs.

a Western blot confirming Mlp1-specific immunoprecipitation from *T. thermophila* using an affinity purified rabbit anti-Mlp1 antibody and a rabbit isotype immunoglobin G (IgG) control tRNAs (n=3 biologically independent samples). The Mlp1-specific antibody was used for both ribonucleoprotein-immunoprecipitation (RNP-IP) and western blotting. The heavy chain (50 kDa) of the antibodies was detected with the secondary anti-rabbit antibody and shown as IgG. Loading control: β-actin. **b,c** Next generation sequencing data of tRNAs split by tRNA isotypes for Mlp1 (n=3 biologically independent samples) (**b**) and hLa from Gogakos *et al*. (n=2 biologically independent samples) (**c**). Fold enrichment is shown as the log2 transformed ratio between Mlp1- or hLa-immunoprecipitated tRNAs and input tRNAs. The small horizontal line represents the average value between replicates. Both Mlp1 and hLa have a higher binding affinity for pre-tRNAs compared to mature tRNAs. **d,e** Binding curves from EMSAs (**d**) and corresponding native gels (**e**) comparing binding affinity of Mlp1 between ^{32}P-labeled pre-tRNA containing 5'- and 3'-extensions or 5'- or 3'-extensions and mature tRNA for Leu^{AAG} and Gln^{UUA} (n=3 independent experiments). The highest binding affinity is found for 5'- and 3'-end containing pre-tRNAs. See **Supplementary Table S1** for Kd quantifications. Source data are provided as a Source Data file.

S3

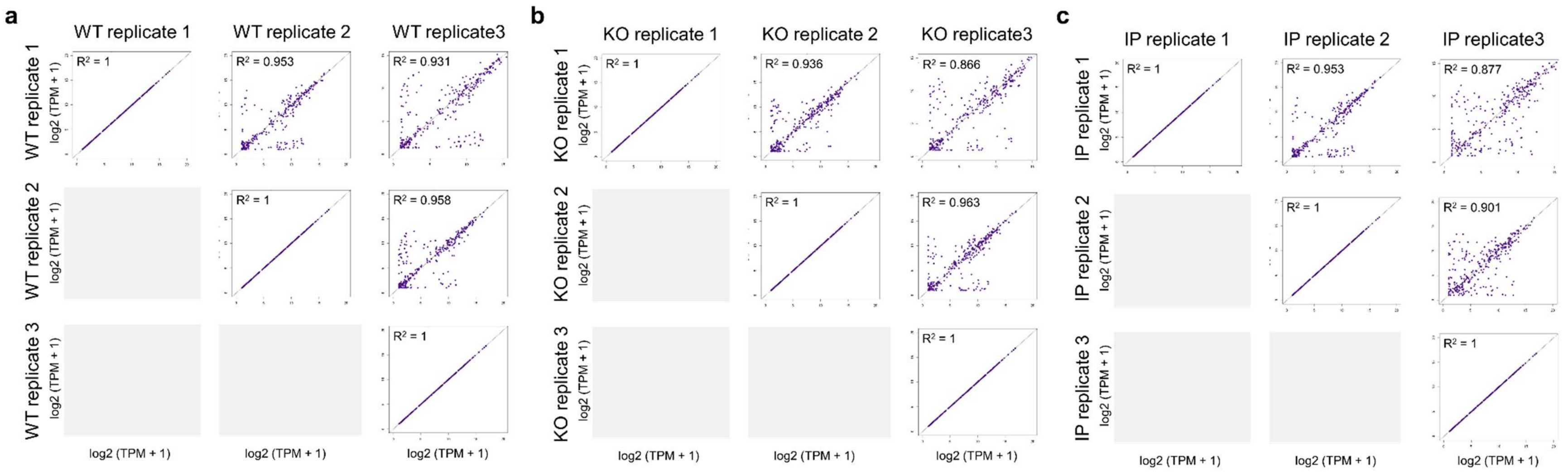

Supplementary Figure S3. TGIRT-sequencing replicates reproducibility determined by correlation plots.
a-c Biological replicates represented as correlation plots following removal of low count reads. Comparison between replicates for tRNA TPM from wild type (WT) (**a**), partial Mlp1 knockout (KO) (**b**), and Mlp1-associated (**c**) samples. R^2 was calculated using the cor function in R. TPM: transcripts per million.

S4

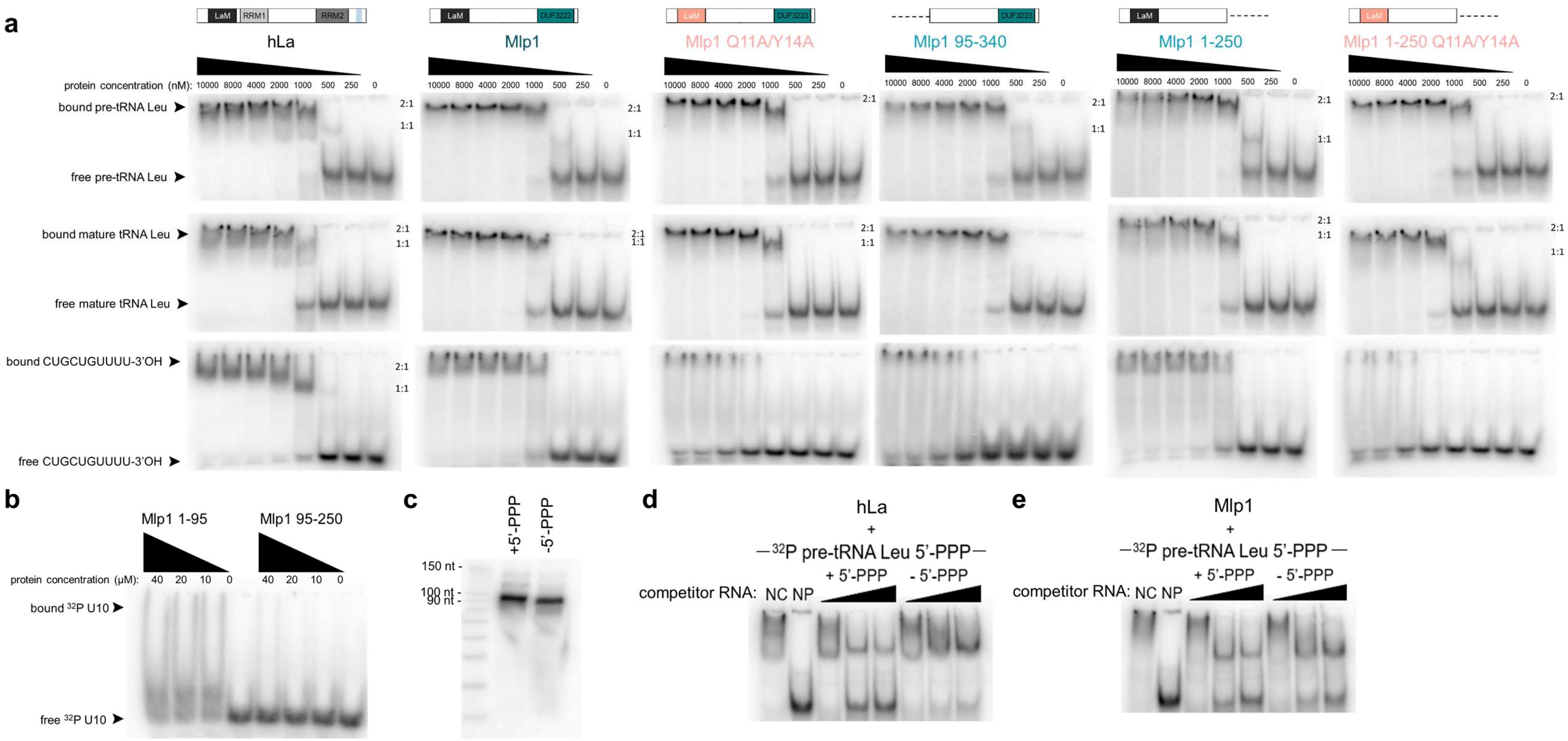

Supplementary Figure S4. Mlp1 binding to typical La protein target RNAs.
a Native gels comparing binding to ^{32}P-labeled pre-tRNAs, mature tRNAs and a 3'-trailer sequence CUGCUGUUUU-3'OH (n≥2 independent experiments). Unbound RNA is found at the bottom of the gel, while protein-RNA complexes shift upwards. The numbering on the side of the gels indicate binding events. A single protein bound to the RNA is indicated at 1:1 and two proteins bound to the same RNA is denoted at 2:1. **b** Native gels comparing binding to ^{32}P-labeled U10 RNA for mutants Mlp1 1-95 (LaM only) and Mlp1 95-250 (middle domain only) (n=2 independent experiments). **c** Denaturing gel of *in vitro* transcribed ^{32}P-labeled 5'-leader containing, 3'-trailer processed pre-tRNAs produced side-by-side as unlabelled-competitor pre-tRNAs (lacking ^{32}P-labeled labeling) used in (**d,e**) to confirm successful removal of the 5'-triphosphate group (n=1 independent experiment). **d,e** Native gels comparing unlabelled-competitors 5'-triphosphate containing pre-tRNA (+5'PPP) and dephosphorylated pre-tRNA (-5'PPP) for ^{32}P-labeled +5'PPP binding on hLa (**d**) or Mlp1 (**e**) (n=2 independent experiments). Competition is seen as a decrease in protein-RNA complex and increase in unbound RNA. NP: no protein lane, NC: no unlabeled competitor lane.

S5

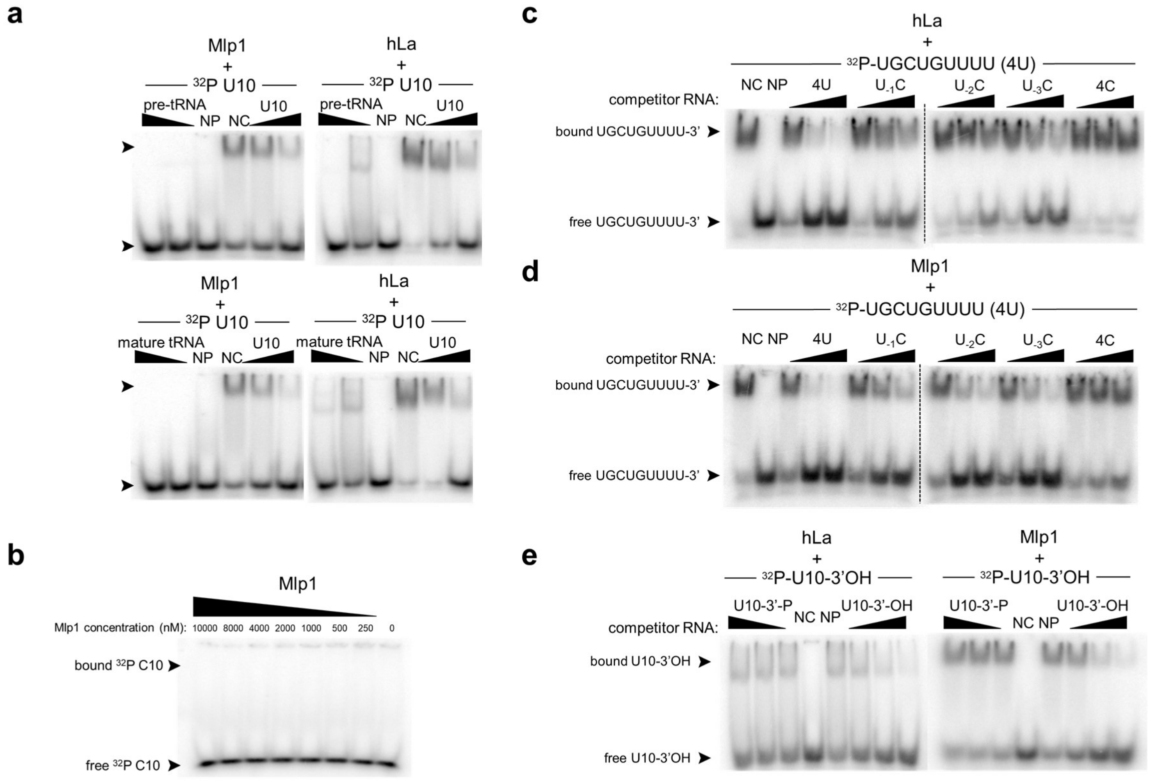

Supplementary Figure S5. Mlp1 binding to uridylate RNA does not discriminate between the position of the uridylate.

a Native gels comparing unlabelled-competitors U10 (positive control), pre-tRNA and mature tRNA for ^{32}P-labeled U10 binding (Mlp1 U10: n=4, tRNA: n=2, hLa U10: n=2, tRNA: n=1 independent experiments). Competition is seen as a decrease in protein-RNA complex and increase in unbound RNA. NP: no protein lane, NC: no competitor lane. **b** Native gel from EMSA for Mlp1 and ^{32}P-labeled C10 (n=2 independent experiments). **c,d** Native gels comparing unlabelled-competitors CUGCUGUUUU (4U), CUGCUGUUUC ($U_{-1}C$), CUGCUGUUCU ($U_{-2}C$), CUGCUGUCUU ($U_{-3}C$) and CUGCUGCCCC (4C) for ^{32}P-labeled 4U wild type RNA binding on hLa (**c**) or Mlp1 (**d**) (n=2 independent experiments). Competition is seen as a decrease in protein-RNA complex and increase in unbound RNA. NP: no protein lane, NC: no competitor lane. **e** Native gels comparing a 3'-phosphorylated substrate (U10-3'-P) and normal 3'-OH containing substrate (U10-3'OH) for binding of ^{32}P-labeled U10-3'-OH. Competition is seen as a decrease in protein-RNA complex and increase in unbound RNA (n=2 independent experiments). No competition was observed with the phosphorylated target for either Mlp1 or hLa. NP: no protein lane, NC: no competitor lane.

S6

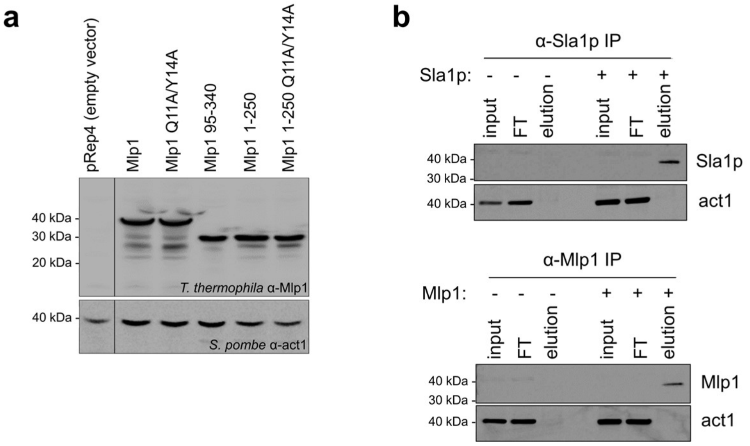

Supplementary Figure S6. tRNA mediated suppression protein expression in *S. pombe*.
a Western blot confirming Mlp1 protein expression levels in *S. pombe* following pRep4 plasmid transformation in the tRNA-mediated suppression assay (n=3 biologically independent samples). Loading control: act1. **b** Western blot confirming immunoprecipitation (IP) of Sla1p and Mlp1 from Sla1p- and Mlp1-transformed *S. pombe* ySH9 tRNA suppressor strains. pRep4 empty vector transformed strains were used as a control for background immunoprecipitation using Sla1p and Mlp1 antibodies (n=2 biologically independent samples). Loading control: act1. FT: flow through. Source data are provided as a Source Data file.

S7

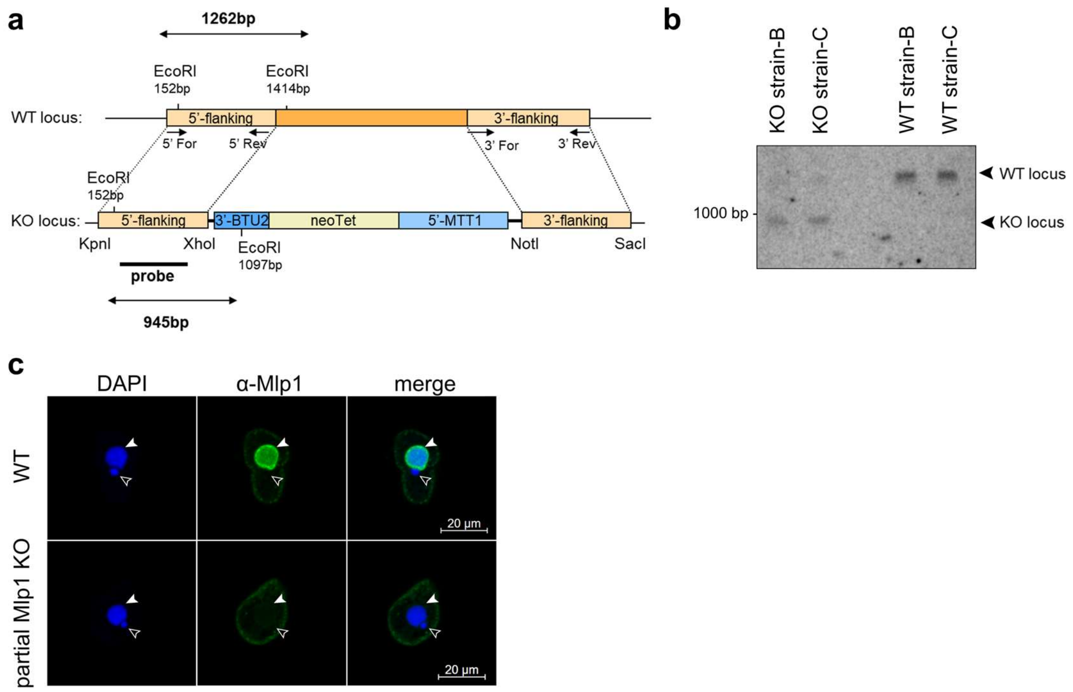

Supplementary Figure S7. Confirmation of the partial Mlp1 *T. thermophila* knockout strain.

a Schematic overview of the *T. thermophila* wild type (WT) locus encoding endogenous Mlp1 and knockout (KO) locus following homologous recombination between 5'- and 3'-flanking regions of the genome with transformation plasmid encoding the neomycin (neoTet) selection marker. Restriction enzymes used for cloning 5'- and 3'-flanking regions for homologous recombination are shown below the KO locus. Genomic DNA digest before Southern blotting was performed with *EcoRI* restriction enzyme generating a 1262 bp fragment for the WT locus and a 945 bp fragment for the KO locus. **b** Southern blot of genomic DNA digested with restriction enzyme *EcoRI* probed with ^{32}P-labeled PCR-generated probe reveals almost complete KO of Mlp1 (see schematic in (**a**)) (n=2 biologically independent samples). **c** Indirect immunofluorescent staining of *T. thermophila* WT and partial Mlp1 KO strains using the polyclonal rabbit anti-Mlp1 antibody. Nuclei were stained using DAPI (n=2 biologically independent samples). Full white arrows are denoting the transcriptionally active macronucleus and empty white arrows show the transcriptionally inactive micronucleus. Source data are provided as a Source Data file.

S8

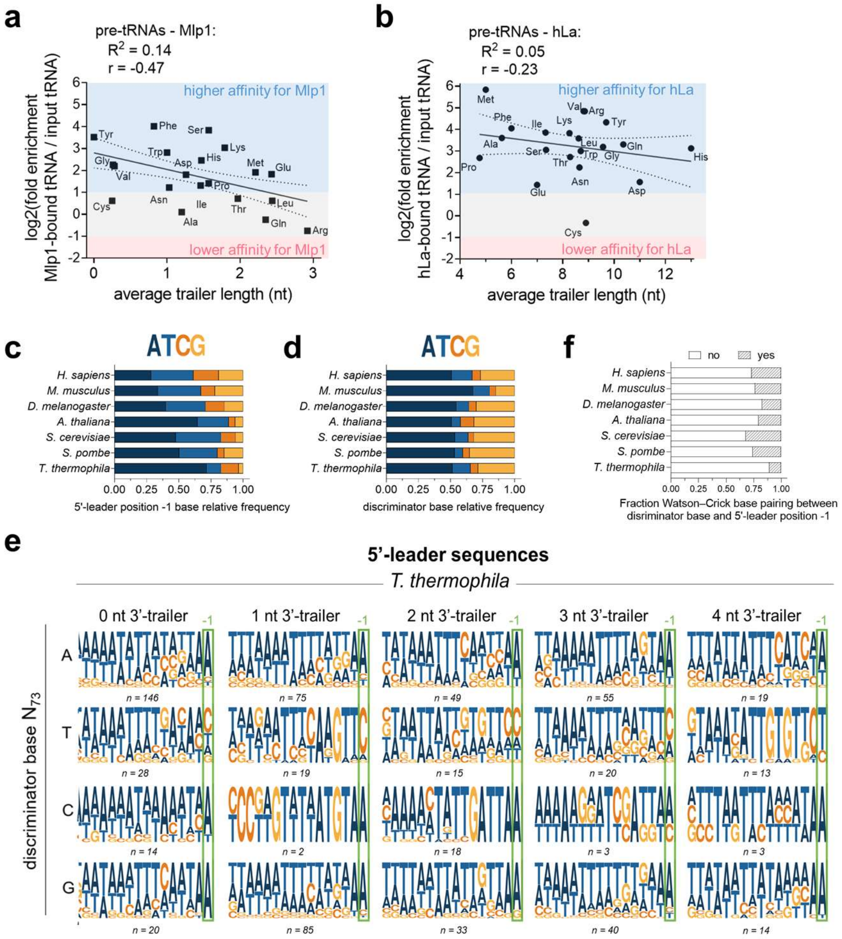

Supplementary Figure S8. The effect of short 3'-trailer sequences on 5'-leader composition and Mlp1 binding affinity.

a,b Next generation sequencing data of Mlp1-bound pre-tRNAs (n=3 biologically independent samples) (**a**) and hLa-bound pre-tRNAs (n=2 biologically independent samples) (**b**) split by tRNA isotypes compared against 3'-trailer lengths. **c** Distribution of the nucleotide at the most 3'-terminal position in the 5'-leader (N_{-1}) of pre-tRNAs from different eukaryotes. *T. thermophila* has a high frequency of adenosines in this position. **d** Distribution of the nucleotide found as the discriminator base (N_{73}) in different eukaryotes. Distribution is equal between different species. **e** Logo analysis of *T. thermophila* 5'-leader sequences split by the discriminator base identity and 3'-trailer length. the most 3'-terminal position in the 5'-leader (N_{-1}) is highlighted with a green box. The number of analyzed pre-tRNAs is shown underneath each logo. **f** Distribution of pre-tRNAs containing a perfect Watson-Crick base pairing between the discriminator base (N_{73}) preceding the 3'-trailer and most 3'-terminal 5'-leader nucleotide (N_{-1}). Source data are provided as a Source Data file.

S9

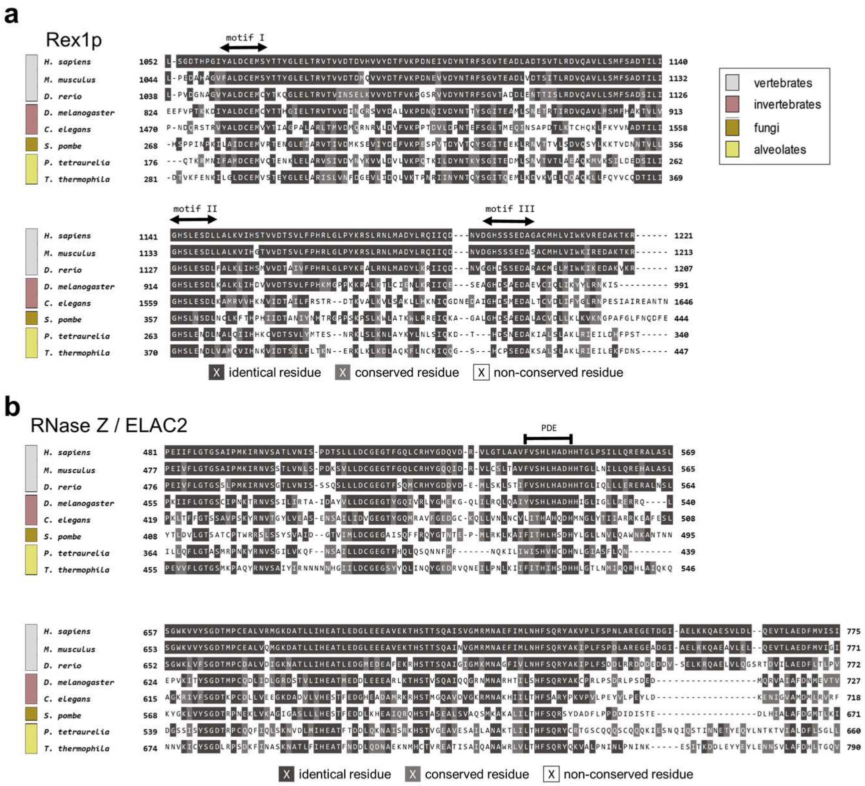

Figure continued on next page

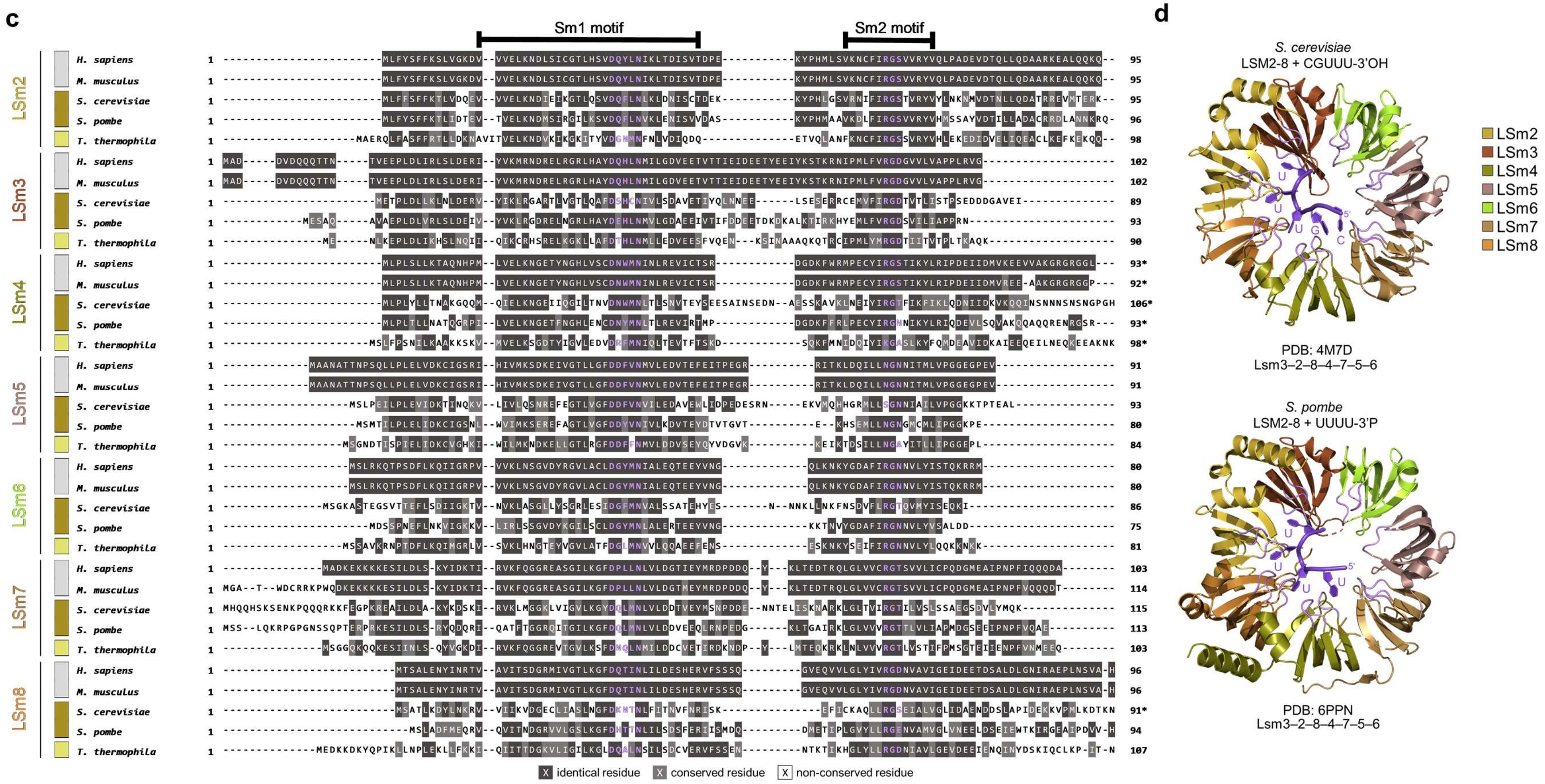

Supplementary Figure S9. Primary sequence alignments of the 3'-exonuclease Rex1p, 3'-endonuclease RNase Z / ELAC2 and LSm2-8 complex from different eukaryotic species.

a-c Primary amino acid alignments from different eukaryotic lineages revealing conservation of the 3'-exonuclease Rex1p (**a**), 3'-endonuclease RNase Z/ELAC2 (**b**) and LSm2-8 complex (**c**). Regions important for Rex1p function are highlighted as motif I, motif II and motif III. The RNase Z catalytic domain is shown as PDE. LSm2-8 complex conserved RNA interacting residues are highlighted in purple and conserved Sm1 and Sm2 motifs are shown. A dark grey background indicates identical residues, light grey conserved residues and white indicates no conservation. **d** High-resolution structures of the Lsm2-8 complex in *S. cerevisiae* (PDB: 4M7D)[5] and *S. pombe* (PDB: 6PPN)[6] in complex with 3'-uridylate RNA shown in dark purple. The conserved RNA interacting residues, highlighted in purple in **c**, are also shown in light purple and are found in the loops.

Supplementary Table S1. Kd values from EMSAs determining binding of Mlp1 to different processed pre-tRNA intermediates and mature tRNA.

K_d: equilibrium dissociation constant, S.D.: standard deviation

	**Leu^{AAG} K_d ± S.D. (nM)**	**Gln^{UUA} K_d ± S.D. (nM)**
pre-tRNA 5' 3'	586 ± 15	687 ± 44
pre-tRNA 3'	658 ± 36	740 ± 40
pre-tRNA 5'	714 ± 21	804 ± 16
mature tRNA	831 ± 33	801 ± 62

Supplementary Table S2. Summary of 3'-trailer length in different eukaryotic species calculated using different RNA polymerase III termination signals.

S.D.: standard deviation, nt: nucleotides

	Termination signal TTTT or $(T)_4$		Termination signal TTTTT or $(T)_5$		Termination signal TTTTTT or $(T)_6$		Termination signal TTTTTT or $(T)_7$	
**Species**	**Average of 3'-trailer length ± S.D. (nt)**	**Number of tRNAs analysed (3'-trailer lengths with >20 nt excluded)**	**Average of 3'-trailer length ± S.D. (nt)**	**Number of tRNAs analysed (3'-trailer lengths with >20 nt excluded)**	**Average of 3'-trailer length ± S.D. (nt)**	**Number of tRNAs analysed (3'-trailer lengths with >20 nt excluded)**	**Average of 3'-trailer length ± S.D. (nt)**	**Number of tRNAs analysed (3'-trailer lengths with >20 nt excluded)**
H. sapiens	8.1 ± 3.7	412	8.4 ± 3.6	140	8.2 ± 3.9	65	7.4 ± 3.7	45
M. musculus	7.9 ± 3.6	343	7.9 ± 3.5	141	7.9 ± 3.4	55	7.6 ± 3.0	39
D. melanogaster	8.5 ± 4.0	247	8.2 ± 3.9	214	8.1 ± 3.9	162	7.1 ± 3.4	79
A. thaliana	4.6 ± 4.3	532	4.7 ± 4.3	441	5.2 ± 4.4	251	4.9 ± 4.1	133
S. cerevisiae	3.0 ± 2.6	269	3.5 ± 3.0	266	3.6 ± 3.2	214	3.9 ± 3.6	132
S. pombe	3.3 ± 2.8	162	4.3 ± 3.8	154	3.9 ± 3.7	86	4.2 ± 4.5	36
T. thermophila	1.5 ± 1.5	686	1.7 ± 1.9	674	2.2 ± 2.9	545	2.5 ± 3.3	312

Supplementary Table S3. **NCBI accession numbers of primary amino acid sequences used for conservation analysis.**

		La	Rex1p	RNase Z / ELAC2	LSm2	LSm3
**Vertebrates**	*Homo sapiens*	NP_003133.1	NP_065746.3	NP_060597.4	NP_067000.1	NP_055278.1
	Mus musculus	NP_033304.1	NP_080128.2	NP_001349912.1	NP_001103571.1	NP_080585.1
	Xenopus laevis	NP_001081021.1				
	Danio rerio	NP_955841	NP_001119888.1	NP_001243133.1		
**Invertebrates**	*Caenorhabditis elegans*	NP_491411.1	NP_498135.2	NP_001023110.1		
	Drosophila melanogaster	NP_477014.1	NP_001034073.1	NP_724916.1		
**Amoebozoa**	*Dictyostelium discoideum*	XP_640625.1				
**Kinetoplastid**	*Trypanosoma brucei*	XP_822491.1				
**Embryophytes**	*Arabidopsis thaliana*	NP_567904.1<br>NP_178106.2				
**Chlorophytes**	*Physcomitrella patens*	XP_024359573.1				
	Chlamydomonas reinhardtii	XP_042914771.1				
**Fungi**	*Schizosaccharomyces pombe*	NP_593315.1	NP_594627.2	NP_595514.1	NP_588459.1	NP_595747.1
	Saccharomyces cerevisiae	NP_010232.1			NP_009527.1	NP_013543.3
**Alveolates**	*Paramecium tetraurelia*	XP_001439258.1	XP_001435945.1	XP_001346883.1		
	Tetrahymena thermophila	XP_001019287.2	XP_001033374.2	XP_001031902.2	XP_012654399	XP_012656392.1

table continued on next page

		LSm4	LSm5	LSm6	LSm7	LSm8
**Vertebrates**	*Homo sapiens*	NP_036453.1	NP_036454.1	NP_009011.1	NP_057283.1	NP_057284.1
	Mus musculus	NP_056631.2	NP_079796.1	NP_001177933.1	NP_001346078.1	NP_598700.1
	Xenopus laevis					
	Danio rerio					
**Invertebrates**	*Caenorhabditis elegans*					
	Drosophila melanogaster					
**Amoebozoa**	*Dictyostelium discoideum*					
**Kinetoplastid**	*Trypanosoma brucei*					
**Embryophytes**	*Arabidopsis thaliana*					
**Chlorophytes**	*Physcomitrella patens*					
	Chlamydomonas reinhardtii					
**Fungi**	*Schizosaccharomyces pombe*	NP_001342832.1	NP_596373.1	NP_594380.1	NP_588340.1	NP_588509.1
	Saccharomyces cerevisiae	NP_011037.3	NP_011073.1	NP_010666.2	NP_014252.2	NP_012556.2
**Alveolates**	*Paramecium tetraurelia*					
	Tetrahymena thermophila	XP_001008519.2	XP_012655775.1	XP_012653392.1	XP_001031297.1	XP_001470817.1

Supplementary References

1. Kotik-Kogan, O., Valentine, E. R., Sanfelice, D., Conte, M. R. & Curry, S. Structural Analysis Reveals Conformational Plasticity in the Recognition of RNA 3′ Ends by the Human La Protein. *Structure* **16**, 852–862 (2008).
2. Dong, G., Chakshusmathi, G., Wolin, S. L. & Reinisch, K. M. Structure of the La motif: A winged helix domain mediates RNA binding via a conserved aromatic patch. *EMBO J.* **23**, 1000–1007 (2004).
3. Apostolidi, M. *et al.* 1H, 15N, 13C assignment and secondary structure determination of two domains of La protein from D. discoideum. *Biomol. NMR Assign.* **8**, 47–51 (2014).
4. Zheng, W. *et al.* LOMETS2: improved meta-threading server for fold-recognition and structure-based function annotation for distant-homology proteins. *Nucleic Acids Res.* **47**, W429–W436 (2019).
5. Zhou, L. *et al.* Crystal structures of the Lsm complex bound to the 3′ end sequence of U6 small nuclear RNA. *Nature* **506**, 116–120 (2014).
6. Montemayor, E. J. *et al.* Molecular basis for the distinct cellular functions of the Lsm1–7 and Lsm2–8 complexes. *RNA* **26**, 1400–1413 (2020).
