# Supplementary material for: Altered tRNA processing is linked to a distinct and unusual La protein in Tetrahymena thermophila
Source: Nat Commun. 2022 Nov 28;13:7332. doi: 10.1038/s41467-022-34796-3 (PMC9705548; doi:10.1038/s41467-022-34796-3)
Supplement: Supplementary file 3 — Description of Additional Supplementary Files [file 41467_2022_34796_MOESM3_ESM.pdf]

Description of Additional Supplementary Files

File Name: Supplementary Data 1
Description: **Number of tRNA genes encoded in the genome of different eukaryotic species for each isotype (top) and isoacceptor (bottom).** The number of tRNA genes was obtained by counting the number of entries for every tRNA isotype and anticodon from the Genomic tRNA Database (GtRNAdb) and from the UCSC Genome Browser for *T. thermophila* in **Supplementary Data 2**. NNN anticodons were excluded from the analysis.

File Name: Supplementary Data 2
Description: **Sequence information for pre-tRNA and mature tRNAs from different eukaryotic species.** The genomic sequences for *H. sapiens, M. musculus, D. melanogaster, A. thaliana, S. Saccharomyces cerevisiae* and *S. pombe* were obtained from the Genomic tRNA Database (GtRNAdb) and *T. thermophila* from the UCSC Genome Browser.

File Name: Supplementary Data 3
Description: **List of oligonucleotides used in this study.**

File Name: Supplementary Data 4
Description: **TGIRT-sequencing normalized tRNA counts from *T. thermophila* samples.** Next generation sequencing tRNA counts are shown as transcripts per million (TPM). Biological triplicates were analysed. Mlp1 IPx: size-excluded Mlp1-associated RNAs following ribonucleoprotein-immunoprecipitation, WTx: size-excluded total RNA from wild type (WT) strain, Mlp1_KOx: size-excluded total RNA from the partial Mlp1 knockout (KO) strain. Mlp1 WT and Mlp1_IP are paired samples obtained from the same biological replicate. Relatively lowly expressed transcripts are shown in grey (TPM < 1). Correlation plots for biological replicates are shown in **Supplementary Fig. S3**

File Name: Supplementary Data 5
Description**: Input sequences for custom Python script to obtain raw counts for 3'-U ending and 3'-CCA ending tRNAs from raw .fastq files.** *T. thermophila* tRNA sequences were screened for unique "fishing" sequences in the 3'-end of the mature tRNA for each isoacceptor (see Summary Table, right) in order to obtain counts for mature tRNAs (ending in -CCA) and pre-tRNAs (ending in different length of -U) (see **Supplementary Data 6 and 7**).

File Name: Supplementary Data 6
Description: **Raw counts for 3'-U ending and 3'-CCA ending tRNAs from raw .fastq files from wild type (WT) input tRNA and Mlp1-immunoprecipitated tRNA (IP).** Data was obtained using a custom python script specifically "fishing" sequence specific reads (see **Supplementary Data 5** for fishing sequences) from the raw .fastq file based on the 3'-ends of the tRNA. Data normalization was done by dividing the raw counts by the scaling factor (sum of transcript counts for each replicate divided by 1,000,000), giving counts per million (CPM) values. Fold enrichment of Mlp1-bound mature tRNAs (3'-CCA) and pre-tRNAs (summation of 3'-U) was calculated by taking the ratio of Mlp1-bound tRNA counts and input WT tRNA counts.

File Name: Supplementary Data 7

Description: **Raw counts for 3'-U ending and 3'-CCA ending tRNAs from raw .fastq files from wild type (WT) and partial Mlp1 knockout (KO) strains.** Data was obtained using a custom python script specifically "fishing" sequence specific reads (see **Supplementary Data 5** for fishing sequences) from the raw .fastq file based on the 3'-ends of the tRNA. Data normalization was done by dividing the raw counts by the scaling factor (sum of transcript counts for each replicate divided by 1,000,000), giving counts per million (CPM) values.
