# Supplementary material for: Altered tRNA processing is linked to a distinct and unusual La protein in Tetrahymena thermophila
Source: Nat Commun. 2022 Nov 28;13:7332. doi: 10.1038/s41467-022-34796-3 (PMC9705548; doi:10.1038/s41467-022-34796-3)
Supplement: Supplementary file 11 — Reporting Summary [file 41467_2022_34796_MOESM11_ESM.pdf]

nature portfolio

Corresponding author(s): Bayfield, MA

Last updated by author(s): 2022/10/14

Reporting Summary

Nature Portfolio wishes to improve the reproducibility of the work that we publish. This form provides structure for consistency and transparency in reporting. For further information on Nature Portfolio policies, see our Editorial Policies and the Editorial Policy Checklist.

Statistics

For all statistical analyses, confirm that the following items are present in the figure legend, table legend, main text, or Methods section.

n/a	Confirmed	
☐	☒	The exact sample size (*n*) for each experimental group/condition, given as a discrete number and unit of measurement
☐	☒	A statement on whether measurements were taken from distinct samples or whether the same sample was measured repeatedly
☐	☒	The statistical test(s) used AND whether they are one- or two-sided<br>*Only common tests should be described solely by name; describe more complex techniques in the Methods section.*
☒	☐	A description of all covariates tested
☐	☒	A description of any assumptions or corrections, such as tests of normality and adjustment for multiple comparisons
☐	☒	A full description of the statistical parameters including central tendency (e.g. means) or other basic estimates (e.g. regression coefficient) AND variation (e.g. standard deviation) or associated estimates of uncertainty (e.g. confidence intervals)
☐	☒	For null hypothesis testing, the test statistic (e.g. *F*, *t*, *r*) with confidence intervals, effect sizes, degrees of freedom and *P* value noted<br>*Give P values as exact values whenever suitable.*
☒	☐	For Bayesian analysis, information on the choice of priors and Markov chain Monte Carlo settings
☒	☐	For hierarchical and complex designs, identification of the appropriate level for tests and full reporting of outcomes
☒	☐	Estimates of effect sizes (e.g. Cohen's *d*, Pearson's *r*), indicating how they were calculated

Our web collection on statistics for biologists contains articles on many of the points above.

Software and code

Policy information about availability of computer code

Data collection	Python v3.9.1<br>SmartBLAST (NCBI)
Data analysis	Trimmomatic v0.36; FastQC v0.11.5; STAR v2.6.1a; CoCo v0.2.5.p1; R package DESeq2 v3.15; conda v4.12; R package pheatmap v1.0.12<br>R package RColorBrewer ;GraphPad Prism v9.0 ;Clustal Omega (EMBL-EBI); PyMOL v2.4.1<br><br>https://github.com/etiennefc/t_thermophila_RNA_Seq.git<br>https://github.com/brunohenderyckx/trna-database-scraper

For manuscripts utilizing custom algorithms or software that are central to the research but not yet described in published literature, software must be made available to editors and reviewers. We strongly encourage code deposition in a community repository (e.g. GitHub). See the Nature Portfolio guidelines for submitting code & software for further information.

Data

Policy information about availability of data

All manuscripts must include a data availability statement. This statement should provide the following information, where applicable:

- Accession codes, unique identifiers, or web links for publicly available datasets
- A description of any restrictions on data availability
- For clinical datasets or third party data, please ensure that the statement adheres to our policy

Data Availability Statement: TGIRT-sequencing has been deposited to the Gene Expression Omnibus (GEO) under the accession number GSE199642. Previously published PDBs used in this study: https://www.rcsb.org/structure/2VOD; https://www.rcsb.org/structure/6PNN; https://www.rcsb.org/structure/1S29; https://www.rcsb.org/structure/2M5W; https://www.rcsb.org/structure/2VOD; https://www.rcsb.org/structure/4M7D. NCBI Mlp1 protein sequence: https://www.ncbi.nlm.nih.gov/protein/XP_001019287.2/; MLP1 reference gene: http://ciliate.org/index.php/feature/details/TTHERM_00384860

Human research participants

Policy information about studies involving human research participants and Sex and Gender in Research.

Reporting on sex and gender	N/A
Population characteristics	N/A
Recruitment	N/A
Ethics oversight	N/A

Note that full information on the approval of the study protocol must also be provided in the manuscript.

Field-specific reporting

Please select the one below that is the best fit for your research. If you are not sure, read the appropriate sections before making your selection.

☒ Life sciences ☐ Behavioural & social sciences ☐ Ecological, evolutionary & environmental sciences

For a reference copy of the document with all sections, see nature.com/documents/nr-reporting-summary-flat.pdf

Life sciences study design

All studies must disclose on these points even when the disclosure is negative.

Sample size	Samples were performed generally in triplicate except where stated otherwise. Sample sizes were generally chosen to be triplicate except where the relevant conclusion was clearly apparent from fewer trials and where statistical significance was not essential for the conclusions drawn. "n" is specified in the manuscript for each relevant experiment. All attempts were successful. There were no experiments that were not able to be reproduced.
Data exclusions	No data were excluded
Replication	Samples were performed in triplicate except where stated otherwise.
Randomization	Randomization was not relevant to the study design. There were no experiments in which sampling was performed.
Blinding	Blinding was not relevant to the study. There were no experiments where sampling was performed. Experiments included positive and negative controls or made reference to previously established or published comparators.

Reporting for specific materials, systems and methods

We require information from authors about some types of materials, experimental systems and methods used in many studies. Here, indicate whether each material, system or method listed is relevant to your study. If you are not sure if a list item applies to your research, read the appropriate section before selecting a response.

Materials & experimental systems

n/a	Involved in the study
☐	☒ Antibodies
☒	☐ Eukaryotic cell lines
☒	☐ Palaeontology and archaeology
☐	☒ Animals and other organisms
☒	☐ Clinical data
☒	☐ Dual use research of concern

Methods

n/a	Involved in the study
☒	☐ ChIP-seq
☒	☐ Flow cytometry
☒	☐ MRI-based neuroimaging

Antibodies

Antibodies used	Primary antibodies used in this study: mouse anti-beta actin (abcam ab8224), rabbit anti-histone H3 (abcam ab1791), mouse anti-His (abcam ab18184) and affinity purified antibodies (ThermoFisher Scientific – Custom Antibodies) rabbit anti-Mlp1 and rabbit anti-Sla1p. Secondary HRP-coupled antibodies used in this study: goat anti-rabbit IgG (Cell Signaling Technology 7074) and horse anti-mouse IgG (Cell Signaling Technology 7076). Relevant dilutions are provided in manuscript.
Validation	anti-Mlp1 and anti-Sla1 custom polyclonals were validated against recombinant protein as well as knockdown/knockout of relevant genes in host organisms (Mlp1: Figure 5A; Sla1: data not shown). Validation for purchased antibodies provided at manufacturer's website: Anti-Actin: https://www.abcam.com/beta-actin-antibody-mabcam-8224-loading-control-ab8224.html, datasheet_8224.pdf; Anti-histone H3: https://www.abcam.com/histone-h3-antibody-nuclear-marker-and-chip-grade-ab1791.html, datasheet_1791.pdf; Anti-His: https://www.abcam.com/6x-his-tag-antibody-hish8-ab18184.html, datasheet_18184.pdf.

Animals and other research organisms

Policy information about studies involving animals; ARRIVE guidelines recommended for reporting animal research, and Sex and Gender in Research

Laboratory animals	N/A
Wild animals	N/A
Reporting on sex	Research was performed in unicellular ciliate for which sex is not a research consideration
Field-collected samples	N/A
Ethics oversight	N/A

Note that full information on the approval of the study protocol must also be provided in the manuscript.
